# Effect of Various Surface Treatments on the Performance of Jute Fibers Filled Natural Rubber (NR) Composites

**DOI:** 10.3390/polym12020369

**Published:** 2020-02-07

**Authors:** Kumarjyoti Roy, Subhas Chandra Debnath, Lazaros Tzounis, Aphiwat Pongwisuthiruchte, Pranut Potiyaraj

**Affiliations:** 1 Department of Materials Science, Faculty of Science, Chulalongkorn University, Bangkok 10330, Thailand; kukumarjyotiroy@gmail.com (K.R.); apw.pongwisuthiruchte@gmail.com (A.P.); 2 Department of Chemistry, University of Kalyani, Kalyani, Nadia 741235, India; scd@klyuniv.ac.in; 3 Department of Materials Science & Engineering, University of Ioannina, 45110 Ioannina, Greece; latzounis@gmail.com; 4 Center of Excellence on Petrochemical and Materials Technology, Chulalongkorn University, Bangkok 10330, Thailand

**Keywords:** rubber, short jute fibers, surface treatments, mechanical properties, scanning electron microscopy

## Abstract

In the present study, the suitability of various chemical treatments to improve the performance of jute fibers (JFs) filled natural rubber (NR) composites was explored. The surface of JFs was modified by three different surface treatments, namely, alkali treatment, combined alkali/stearic acid treatment and combined alkali/silane treatment. Surface modified JFs were characterized by X-ray diffraction (XRD) pattern, Fourier transform infrared (FTIR) spectroscopy and field emission scanning electron microscopy (FESEM). The reinforcing effect of untreated and surface treated JFs in NR composites was comparatively evaluated in terms of cure, mechanical, morphological and thermal properties. Combined alkali/silane treated JFs filled NR composite showed considerably higher torque difference, tensile modulus, hardness and tensile strength as compared to either untreated or other surface treated JFs filled NR systems. A crosslink density measurement suggested effective rubber-fibers interaction in combined alkali/silane treated JFs filled NR composite. Morphological analysis confirmed the improvement in the interfacial bonding between NR matrix and JFs due to combined alkali/silane treatment allowing an efficient “stress-transfer” mechanism. As a whole, combined alkali/silane treatment was found to be most efficient surface treatment method to develop strong interfacial adhesion between NR matrix and JFs.

## 1. Introduction

Short fiber reinforced rubber composites are commonly used for the manufacturing of several industrial products such as hoses, seals, tire treads, V-belts, complex-shaped goods, etc. [1,2]. The overall performances of short fiber reinforced rubber composite are closely related to some factors like strong adhesion between rubber and fibers, aspect ratio of fibers, degree of dispersion of fibers within the rubber matrix and control of fibers orientation [2,3]. Natural fibers are bio-based renewable materials with some interesting features like high specific strength, low self-weight, unrestricted formability and resistance to corrosion [4,5]. Among the various non-petroleum based materials, natural occurring jute fibers (JFs) are one of the most promising alternatives to traditional petroleum based fillers for the development of environmentally friendly rubber composites.

In the last two decades, due to upward environmental awareness, there is an increasing demand for the development of natural fiber based green and sustainable rubber composites. Many researchers reported the designing of advanced rubber composites based on different types of natural fiber such as short jute [2,6,7,8,9], bamboo [10,11], short coir [12,13,14], sisal [15,16], oil palm [15,16,17], kenaf [18], grass [19,20], hemp [21,22], pineapple leaf [23,24], etc. Compared to petroleum based materials, natural fibers have some additional advantages, i.e., availability, biodegradability, light-weight, low-cost, renewability and non-toxic nature [9,25]. However, the proper dispersion of short natural fibers in natural rubber (NR) composites is a challenging task due to the poor compatibility between the hydrophilic natural fibers and the hydrophobic rubber matrix.

Among the different natural fibers, jute is a commercially cheap lignocellulosic fiber with large cellulose content [7,25]. Initially, Murty et al. introduced short JFs as new reinforcing material in rubber composites [6]. In recent years, very few research articles have been published regarding the use of short JFs as a green reinforcing filler to improve the performance of NR compounds [2,8,9]. Surface treatment of JFs is the key strategy to enhance the interfacial adhesion between hydrophilic jute and hydrophobic NR matrix [8]. Tzounis et al. [9] investigated the effect of carbon nanotube modified JFs (JF-CNT) as “hierarchical” multiscale reinforcements on the mechanical and thermal properties of NR composites. According to the authors, JF-CNT filler had better hydrophobic character than unmodified JFs as filler, while the nanostructured surface of the JFs due to the roughness endowed by the CNTs facilitated a mechanical interlocking mechanism between the fiber–matrix components. As a result, JF-CNT filled NR composites exhibited considerably higher tensile properties and thermal stability as compared to those of unmodified JFs filled NR samples at same filler loading level. Recently, Roy et al. [2] reported on the role of stearic acid modified nanoclay (SANC) on the cure, mechanical and thermal properties of alkali treated JFs filled NR composites. Actually, SANC was able to increase the hydrophobic character of JFs due to the hydrogen bonding interaction between the carboxyl group of SANC and surface hydroxyl groups of JFs. Thus, the tensile strength and storage modulus of JFs filled NR composites were greatly improved in presence of SANC. Nevertheless, the research work concerning the use of JFs as green and natural occurring filler for the development of both environmental and industrial friendly rubber composites is in the preliminary stage.

The main aim of the present study was to achieve a novel concept for the development of high performance and low-cost natural JFs reinforced NR composites. The performance of JFs filled NR composites was closely connected to the “engineered” and by design adhesive interface between rubber and fibers in this study. More precisely, the JFs surface modification endowed enhanced interfacial compatibility between NR and JFs with an enhanced interfacial strength as supported by the chemical interaction mechanism that have been illustrated as well as the SEM fracture surfaces. Undoubtedly, the present study demonstrates a facile, versatile, scalable and unique protocol to develop natural fibers based green rubber composites making use of surface science and technology.

## 2. Materials and Methods

### 2.1. Materials

NR (RMA-1X), zinc oxide (surface area 5–6 m^2^/g, Merck, Kenilworth, NJ, USA), stearic acid (LobaChemie, Mumbai, India), sulfur (LobaChemie, Mumbai, India), sodium hydroxide (Merck) and toluene (Merck) were used as received. Tetra methyl thiuram disulfide (TMTD) was procured from Thailand Rubber Research Institute (Bangkok, Thailand). JFs (TD 4 grade) were obtained from Gloster Jute Mills, Howrah, India. Bis[3-(triethoxysilyl)propyl]tetrasulfide (TESPT) (Sigma-Aldrich, Dorset, UK) were used as received.

### 2.2. Surface Modification of JFs

#### 2.2.1. Alkali Treatment

**Figure 1:**
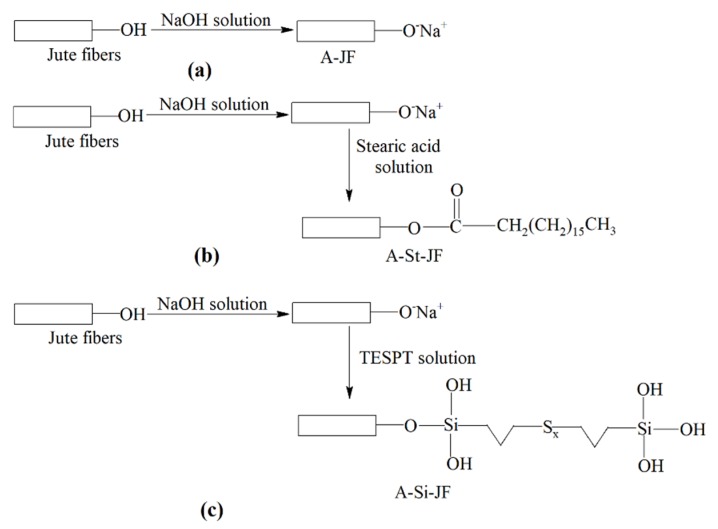
Mechanism of surface modification of jute fibers (JFs) by different chemical treatments, (**a**) alkali treatment, (**b**) combined alkali/stearic acid treatment, (**c**) combined alkali/silane treatment.

At first, short JFs were cut and ground to powder form using a mixer grinder. Then, the JFs were ultrasonicated in 1 wt % aqueous solution of sodium hydroxide (NaOH) for 1 h to eliminate lignin and hemicelluloses. Next, the alkali treated JFs were washed with distilled water followed by neutralization with diluted acetic acid until the fibers surface becomes completely free from the unreacted alkali [9]. Then, the JFs were again washed with distilled water. Finally, the alkali treated JFs were dried in a hot air oven at 70 °C for 24 h. The untreated JFs were designated hereafter as JFun. The alkali treated JFs were also designated as A-JF. The mechanism of alkali surface treatment of JFs is shown in Figure 1a.

#### 2.2.2. Combined Alkali/Stearic Acid Treatment

Initially, 1 wt % stearic acid was added to water and heated to prepare a homogeneous solution. After that, the alkali modified JFs (A-JF) were treated with 1 wt % stearic acid solution in an ultrasonication bath for 1 h. Then, the surface treated JFs were washed with toluene followed by methanol to remove excess unreacted stearic acid. Finally, combined alkali/stearic acid treated JFs were dried in a hot air oven at 70 °C for 24 h. The combined alkali/stearic acid treated JFs were designated as A-St-JF. The mechanism of surface treatment of JFs by combined alkali/stearic acid is shown in Figure 1b.

#### 2.2.3. Combined Alkali/Silane Treatment

At first, 1 wt % solution of silane coupling agent, i.e., TESPT was prepared in water:ethanol mixture (water:ethanol = 40:60). Subsequently, the alkali treated jute fibers (A-JF) were treated with 1 wt % solution of TESPT in an ultrasonication bath for 1 h. Finally, the surface treated JFs were washed with distilled water to remove the unreacted TESPT followed by drying in a hot air oven at 70 °C for 24 h. The combined alkali/silane treated JFs were designated as A-Si-JF. The mechanism of surface treatment of JFs by combined alkali/silane is shown in Figure 1c.

### 2.3. Preparation of NR Composites

Various NR composites were prepared in a two-roll mixing mill according to the formulation shown in Table 1. The compounding of various NR composites was carried out at room temperature. During mixing process, the speed of one roll was kept at 20 rpm and that of the other roll was maintained at 24 rpm to attain the friction ratio of 1:1.2.

### 2.4. Characterization Techniques

X-ray diffraction (XRD) patterns of untreated and surface treated JFs were recorded on Xpertpro-Panalytical X-ray diffractometer (Malvern, UK). Scanning electron microscopy (SEM) images of JFs and tensile fracture surfaces of NR composites were obtained using field emission scanning electron microscopy (FESEM, JEOL, JSM-7610 F, Tokyo, Japan). Fourier transform infrared (FTIR) investigations of untreated and surface treated JFs were performed using Perkin-Elmer L 120-000A spectrometer (Waltham, MA, USA) (*ν*_max_ in cm^−1^) on KBr disks. The cure characteristics like minimum torque (ML), maximum torque (MH), scorch time (*t*_2_) and optimum cure time (*t*_90_) were measured on a moving die rheometer (rheoTech MD+, Model no. A022S, Ajpha Technologies, Akron, OH, USA) at 160 °C. Different compounded NR samples were cured according to their optimum cure time at 160 °C in a hot press. For various NR sheets, the mechanical properties like modulus at 100% (M100) elongation, tensile strength (T.S.) and elongation at break (E.B. in %) were measured using Amsler (Göteborg, Sweden) tensile tester according to ASTM D 412-51 T. Hardness (shore A) of different NR samples was calculated by a Hiroshima Hardness Tester (Aishwarya, Telangana, India) according to ASTM D 2240. Crosslink density values and solvent uptake properties of unfilled and filled NR samples were measured from swelling experiment according to the method given by Roy et al. [26]. Thermo-gravimetric analysis (TGA) was carried out using a TGA instrument (Mettler Toledo, Columbus, OH, USA), TGA/DSC 3+ under nitrogen flow from 50 to 600 °C with a heating rate of 10 °C/min.

## 3. Results and Discussion

### 3.1. Confirmation of Surface Modification of JFs

**Figure 2:**
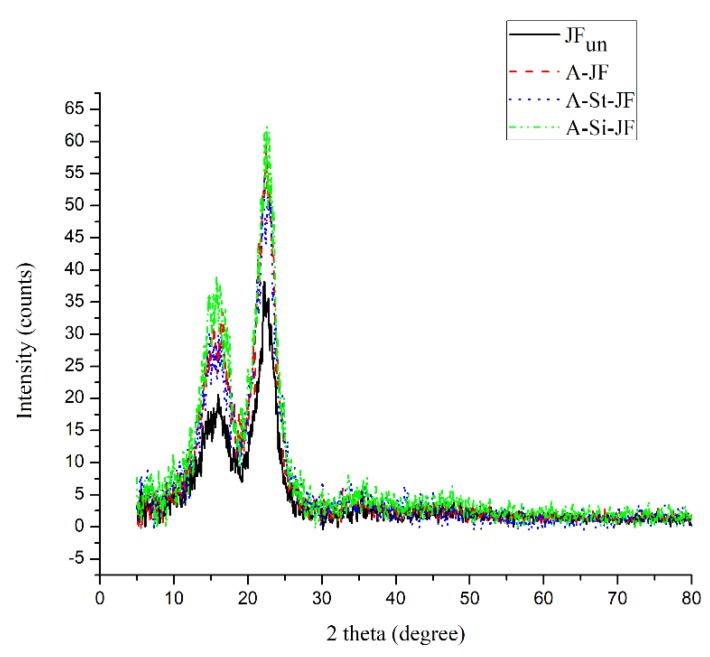
XRD pattern of unmodified and surface modified JFs.

The XRD patterns of untreated and surface treated JFs are represented in Figure 2. In both untreated and surface treated JFs, the common peaks at about 17 and 22.5° were attributed to the (101) and (002) planes of cellulose [27]. Thus, there was no noticeable change in the macromolecular chain structure of JFs due to the surface treatments [27]. However, diffraction peak intensities of surface treated JFs showed clear increment as compared to untreated JFs. This result was due to the increase in the ratio of crystalline cellulose resulting from the removal of amorphous waxy substances after surface treatments [27].

**Figure 3:**
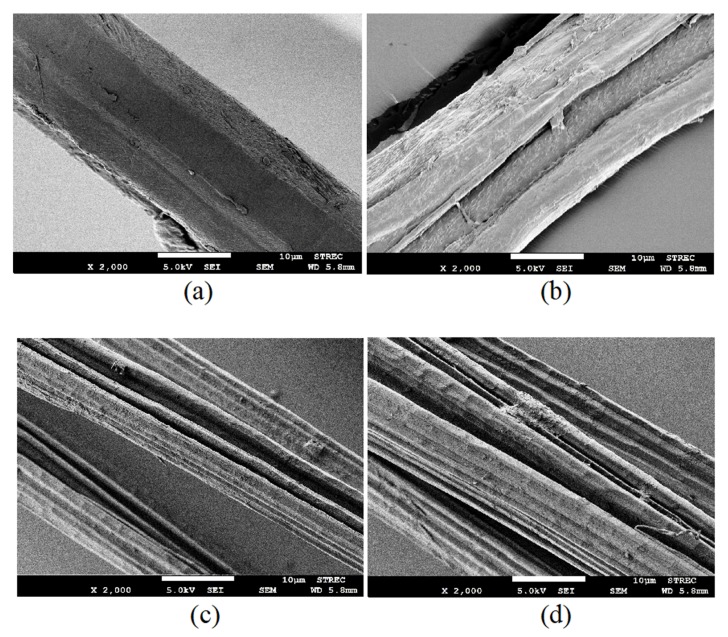
FESEM images of (**a**) JF_un_, (**b**) A-JF, (**c**) A-St-JF and (**d**) A-Si-JF.

The surface morphology of various JFs was examined using FESEM analysis. The FESEM images of untreated and surface treated JFs are presented in Figure 3. As shown in Figure 3a, JFun had smooth surface owing to the presence of waxy substances like lignin and hemicelluloses [27,28]. On the other hand, several grooves were formed along the structure of chemically treated JFs due to the elimination of waxy substances after surface treatments. As a result, all surface modified JFs, namely, A-JF, A-St-JF and A-Si-JF showed higher surface roughness than JFun (Figure 3b–d). The rough surface morphology was the key factor for the enhancement of interfacial strength due to mechanical interlocking between surface treated JFs and rubber matrix [27,28].

**Figure 4:**
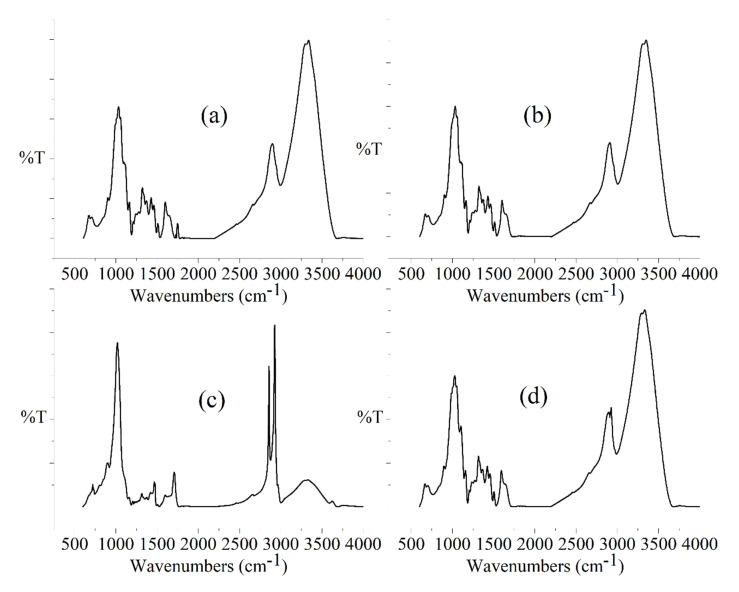
FTIR spectra of (**a**) JF_un_, (**b**) A-JF, (**c**) A-St-JF and (**d**) A-Si-JF.

FTIR spectra of untreated and chemically treated JFs are shown in Figure 4. Both, untreated and surface treated JFs showed common peak at about 900 cm^−1^ due to the C–H bending mode of cellulose [27]. As shown in Figure 4a, there was a significant peak around 1737 cm^−1^ in the FTIR spectrum of JFun due to the C=O stretching vibration resulting from carboxyl and acetyl groups in hemicelluloses. This peak of hemicelluloses disappeared in the FTIR spectra of surface treated JFs. This result indicated the proper removal of waxy substances from JFs due to the surface treatments. As shown in Figure 4c, the FTIR spectrum of A-St-JF showed some characteristics band of stearic acid at about 2923 and 2856 cm^−1^ due to C–H stretching vibration of methylene groups [28]. Some interesting peaks were observed in the FTIR spectrum of A-Si-JF. As shown in Figure 4d, a clear peak at about 2924 cm^−1^ was attributed to the existence of C–H stretching vibration of CH_3_ and CH_2_ groups of TESPT [29]. In addition, the presence of a peak around 1100 cm^−1^ was associated with the formation of silicon-oxygen bond in the combined alkali/silane treated JFs [29,30].

### 3.2. Cure Characteristics

**Figure 5:**
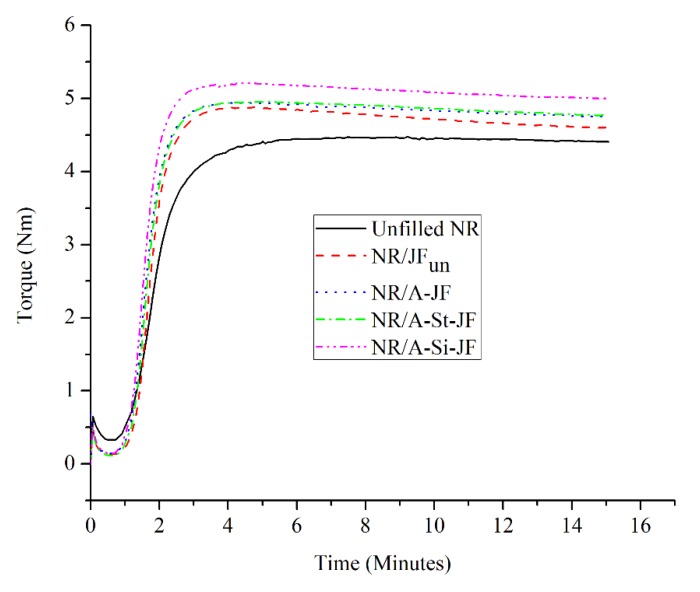
Cure curves of unfilled and JFs filled natural rubber (NR) composites.

The cure curves of unfilled and JFs filled NR composites are represented in Figure 5. Cure characteristics of various NR composites are also displayed separately in Table 2. The value of maximum torque (MH) of NR composites showed considerable increment due to the addition of both untreated and surface treated JFs as filler, which implies the restriction of the mobility of the NR chains in presence of filler [31]. In other words, the increase in the MH value of filled NR compounds was closely related to the increase in the stiffness of NR composites due to the incorporation of both untreated and surface treated JFs. Again, the value of torque difference i.e., the difference between maximum torque (MH) and minimum torque (ML) is the indirect measure of crosslink density for rubber composites [31,32]. As shown in Table 2, both untreated and surface treated JFs were able to increase the torque difference value of NR composites. Thus, there was a successful enrichment in the crosslink density of NR composites in presence of both untreated and surface treated JFs. Among the various filled NR composites, the torque difference value was found to be maximum for NR composite filled with A-Si-JF. This result might be explained by considering the exceptional improvement in the interfacial interaction between NR matrix and JFs after combined alkali/silane treatment [26].

The values of scorch time (*t*_2_) and optimum cure time (*t*_90_) decreased markedly due to the addition of both untreated and chemically treated JFs into the NR matrix. Actually, a rubber sample remains for a greater time on the mixing mill during preparation of filled rubber composites. Thus, for filled NR composites, the decreasing trend of *t*_2_ and *t*_90_ was closely related to the generation of greater amount of heat due to additional friction [10]. NR/A-Si-JF had shorter *t*_2_ and *t*_90_ than other JFs filled NR composites, which is attributed to the optimum dispersion of A-Si-JF within the NR matrix [10].

### 3.3. Mechanical Properties

The mechanical properties of NR composites in the presence of untreated and surface treated JFs are summarized in Table 3. The value of modulus at 100% elongation (M100) of NR composites showed clear increment due to the addition of both untreated and surface treated JFs. However, surface modified JFs were more effective to increase the M100 value of NR composites as compared to unmodified raw JFs. NR/A-Si-JF exhibited highest M100 value among the various JFs filled NR composites. The value of M100 was found to increase by 113% for NR/A-Si-JF system in comparison to an unfilled NR system. This result confirms the outstanding enhancement in the crosslink density of NR/A-Si-JF system due to the excellent interfacial interaction between the NR matrix and A-Si-JF [30]. On the other hand, both untreated and surface treated JFs had a similar effect on the hardness of the NR composites. The hardness of the NR composites increased rapidly in the presence of JFs as filler, which indicates the formation of stiffer NR composites due to the addition of filler materials into the rubber matrix [32]. The variation of hardness followed the same trend as that of the M100 of filled NR composites and NR/A-Si-JF had higher hardness value as compared to other JFs filled NR composites. This might be due to the better crosslink density of NR/A-Si-JF composite than all other filled NR composites.

The tensile strength values of NR composites in presence of various types of JFs are also comparatively depicted in Table 3. The value of tensile strength showed a clear reduction due to incorporation of JFun into the NR matrix, which is attributed to the poor dispersion of untreated hydrophilic JFs within the hydrophobic NR matrix. However, surface modified JFs had slightly positive effect on the tensile strength of NR composites. The tensile strength value suggested the understandable reinforcing effect of various surface treated JFs in NR compounds. This result might be due to the improved dispersion of surface modified JFs within the NR matrix. Further, the tensile strength of NR/A-Si-JF was notably higher as compared to either NR/A-St-JF or NR/A-JF. Thus, combination of alkali and silane treatment was the most effective technique to improve the dispersion level of JFs within NR matrix. The value of elongation at break was lower for various JFs filled NR composites as compared to unfilled NR composite, which is due to the increase of stiffness and brittleness of NR composites in presence of filler [33].

### 3.4. Crosslink Density

**Figure 6:**
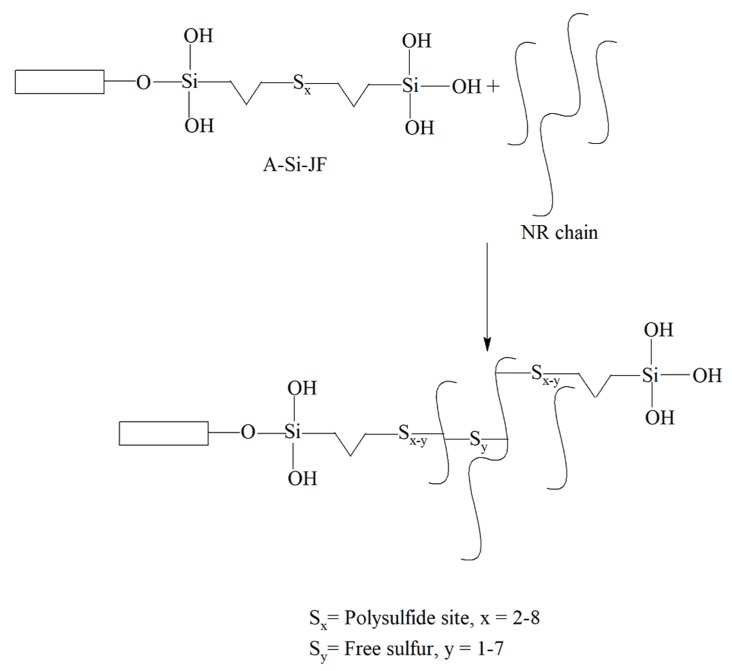
The probable mechanism of cross-linking between A-Si-JF and NR chains.

Crosslink density is a unique parameter, which is closely linked with the cure and mechanical performances of filled rubber composites. The crosslink density values of unfilled and filled NR composites are displayed in Table 3. It was found that surface modified JFs based NR composites exhibited noticeably higher crosslink density as compared to unmodified JFs based NR composite, indicating excellent interfacial interaction between NR matrix and modified JFs. Among the three surface modified JFs, A-Si-JF facilitated greater crosslink density for NR composite as compared to either A-St-JF or A-JF. Therefore, TESPT had a clear role on the crosslink density of A-Si-JF filled NR composite. The plausible mechanism of cross-linking between A-Si-JF and NR chains is schematically illustrated in Figure 6. The variation of crosslink density was found to be in good agreement with the torque difference, hardness and tensile modulus of NR composites.

### 3.5. Morphology of Composite Fracture Surfaces

**Figure 7:**
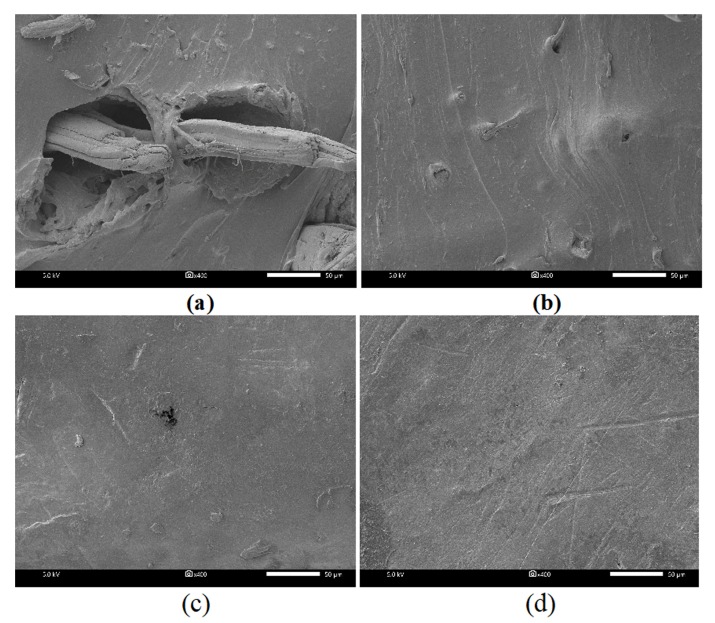
FESEM micrographs of (**a**) NR/JF_un_, (**b**) NR/A-JF, (**c**) NR/A-St-JF and (**d**) NR/A-Si-JF.

Filler dispersion is an important parameter regarding the mechanical performances of filled rubber composites [34]. FESEM analysis was performed to compare the degree of dispersion of untreated and surface treated JFs in NR composites. Figure 7 shows the morphological characteristics of untreated and surface treated JFs filled NR composites fractured surfaces. As shown in Figure 7a, many holes were generated due to fibers pulling out from NR matrix in NR/JFun composite. This was due to the poor interfacial adhesion between hydrophobic NR matrix and hydrophilic JFs. As a result, the tensile strength of NR/JFun composite was lower than the unfilled NR composite. On the other hand, the fracture surfaces of NR/A-JF and NR/A-St-JF showed better wetting and dispersion of JFs within NR matrix (Figure 7b,c). Thus, moderate improvement in the mechanical properties was observed in the cases of NR/A-JF and NR/A-St-JF composites. As shown in Figure 7d, NR/A-Si-JF composite had very smooth and continuous surface, which indicates excellent interfacial adhesion between NR matrix and A-Si-JF. The remarkable enhancement in the tensile strength, modulus and crosslink density was reflected to the efficacious interfacial adhesion between NR matrix and A-Si-JF in NR/A-Si-JF composite.

### 3.6. Solvent Uptake Behaviour of JFs Filled NR Composites

**Figure 8:**
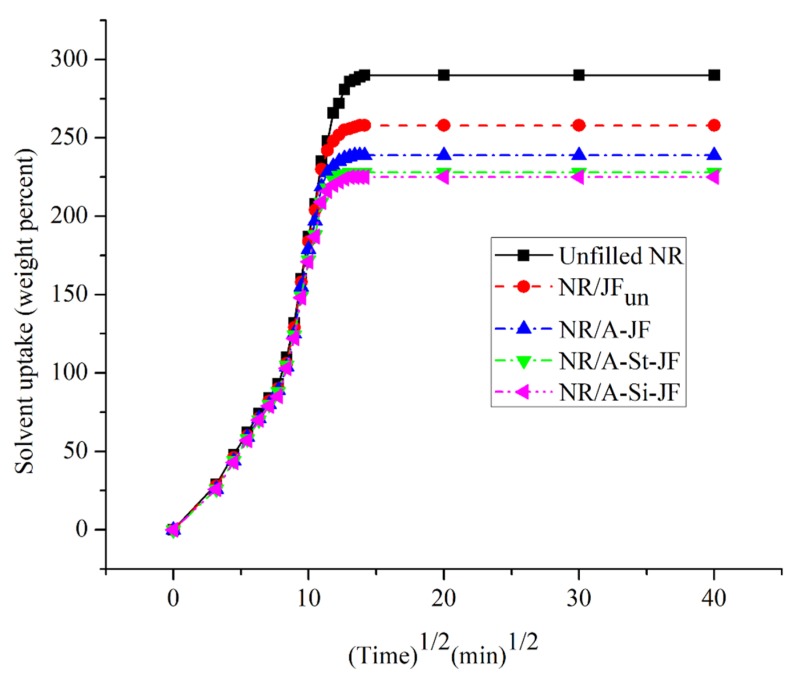
Solvent uptake behavior of unfilled and JFs filled NR composites.

**Figure 9:**
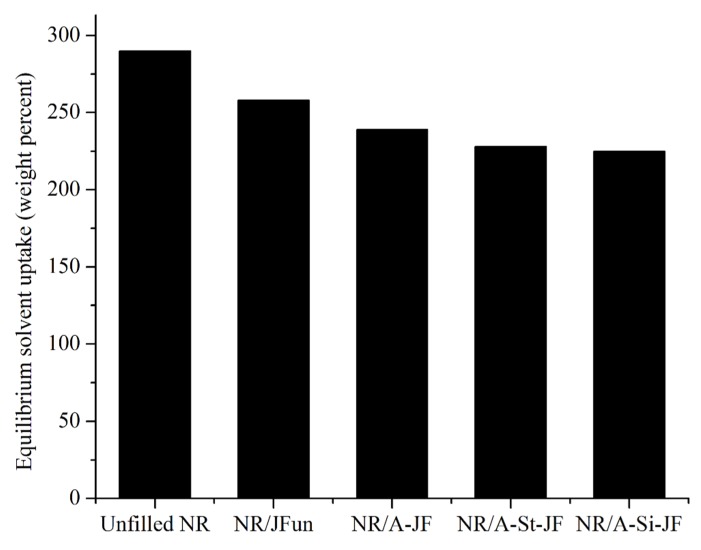
The values of equilibrium solvent uptake of different NR composites.

Solvent uptake behavior provides indirect information about the interaction between cellulose based JFs and NR matrix [26]. The lowering of solvent uptake is related to the good interaction between JFs and NR matrix [26]. The plots of solvent uptake (weight percent) vs. (time) 1/2 of different JFs filled NR composites are illustrated in Figure 8. All the solvent uptake plots showed a similar pattern with speedy solvent uptake at smaller time region. The values of equilibrium solvent uptake (weight percent) of unfilled and JFs filled NR composites are also presented in Figure 9. The values of equilibrium solvent uptake of surface treated JFs filled NR composites were considerably lower as compared to either untreated JFs filled NR composite or unfilled NR composite. Moreover, the lowest value of equilibrium solvent uptake was found for an NR composite filled with A-Si-JF. Hence, the rubber-JFs interaction was improved obviously due to the surface modification of fibers by combined alkali/silane treatment.

### 3.7. Thermal Properties of JFs Filled NR Composites

**Figure 10:**
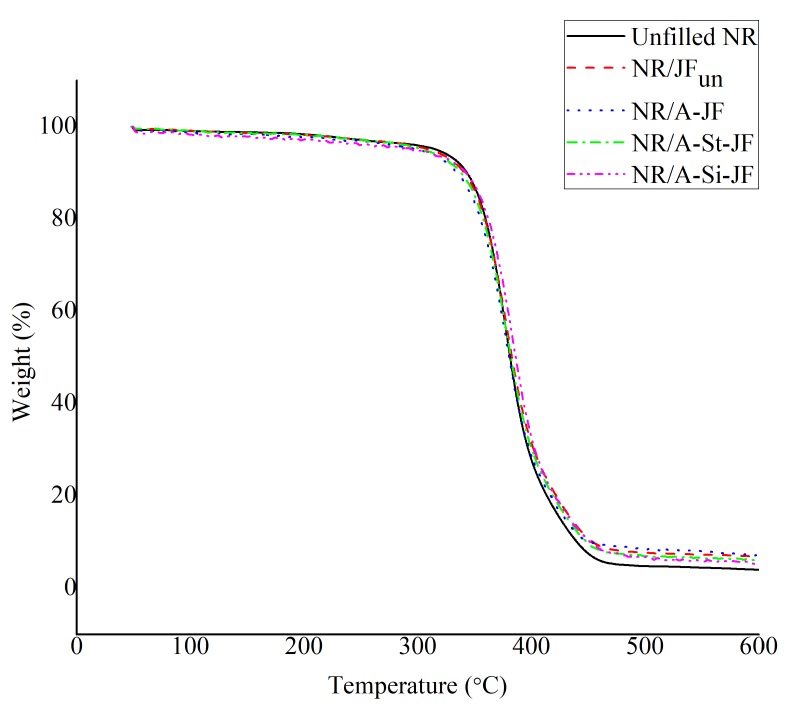
TGA curves of unfilled and JFs filled NR composites.

TGA study was utilized to compare the thermal stability of untreated and surface treated JFs filled NR composites. Different TGA curves of unfilled and filled NR samples are shown in Figure 10. The TGA results of various NR composites are summarized in Table 4. The thermal stabilities of various NR composites were examined in terms of temperature corresponds to 10% weight loss (*T*_10%_), temperature corresponds to 20% weight loss (*T*_20%_), temperature corresponds to 50% weight loss (*T*_50%_) and temperature corresponds to 80% weight loss (*T*_80%_). There was no improvement in the values of *T*_10%_ and *T*_20%_ of NR compounds in the presence of both untreated and surface treated JFs. The NR/A-Si-JF composite showed a little increment in the values of *T*_50%_ and *T*_80%_ as compared to either unfilled NR or NR/JFun composites, which indicates a slight improvement in the thermal stability of NR composite in presence of A-Si-JF. Actually, the mobility of NR chains was restricted in the vicinity of A-Si-JF due to the presence of strong rubber-fibers interaction [35]. Thus, the diffusion of the degradation products from the NR/A-Si-JF system was little bit more difficult as compared to both unfilled NR and NR/JFun systems.

## 4. Conclusions

The main goal of the present study was to elucidate the potential of different surface treated JFs as a non-petroleum based filler for the advancement of commercially serviceable rubber technology. For this purpose, the surface of JFs was modified by using three different chemical approaches, i.e., alkali treatment, combined alkali/stearic acid treatment and combined alkali/silane treatment. The cure characteristics, mechanical, morphological, solvent uptake and thermal properties were comparatively assessed for NR composites filled with untreated and surface treated JFs. The value of the torque difference increased clearly due to the incorporation of both untreated and chemically treated JFs into NR matrix. Among the various JFs filled NR composites, NR/A-Si-JF provided highest value of torque difference. On the other hand, surface modified JFs based NR composites showed better mechanical properties as compared to either unmodified JFs based NR composite or unfilled NR composite. More importantly, the increment of mechanical properties was more predominant in case of NR/A-Si-JF composite as compared to either NR/A-St-JF or NR/A-JF. In addition, this was corroborated by the corresponding morphological analysis demonstrating an outstanding interfacial adhesion between NR matrix and A-Si-JF. The morphological observation was found thus to be in good agreement with the variation of mechanical properties in JFs filled NR composites. Moreover, A-Si-JF offered slightly better thermal stability for NR compounds than untreated JFs. For the first time, A-Si-JF was utilized as a novel green filler in NR based rubber composites. Finally, it could be envisaged that A-Si-JF may find important place as a suitable filler for the progress of commercially viable and environmentally friendly green rubber technology.

## Figures and Tables

**Table 1:** The formulation of studied vulcanizates in parts per hundred parts of rubber (phr).

Ingredients	Compound Designation				
	Unfilled NR	NR/JF_un_	NR/A-JF	NR/A-St-JF	NR/A-Si-JF
NR	100	100	100	100	100
ZnO	5	5	5	5	5
Stearic acid	2	2	2	2	2
TMTD	2.16	2.16	2.16	2.16	2.16
Sulfur	0.5	0.5	0.5	0.5	0.5
JF_un_	-	10	-	-	-
A-JF	-	-	10	-	-
A-St-JF	-	-	-	10	-
A-Si-JF	-	-	-	-	10

**Table 2:** Cure properties of NR composites.

Formulation	Maximum Torque (Nm)	Torque Difference (Nm)	Scorch Time, *t*_2_ (min)	Optimum Cure Time, *t*_90_ (min)
Unfilled NR	4.48	4.07	1.85	3.17
NR/JF_un_	4.88	4.73	1.66	2.48
NR/A-JF	4.94	4.77	1.55	2.34
NR/A-St-JF	4.96	4.78	1.6	2.4
NR/A-Si-JF	5.23	4.98	1.5	2.26

**Table 3:** Mechanical properties of NR composites.

Formulation	M_100_ (MPa)	Hardness (Shore A)	Tensile Strength (MPa)	Elongation at Break (%)	Crosslink Density × 10^5^ (mol cm^−3^)
Unfilled NR	0.83 ± 0.04	47 ± 2	12.07 ± 0.58	835 ± 15	7.21
NR/JF_un_	1.20 ± 0.09	55 ± 2	10.52 ± 0.69	765 ± 15	8.54
NR/A-JF	1.49 ± 0.07	58 ± 1	14.21 ± 0.89	750 ± 20	8.76
NR/A-St-JF	1.63 ± 0.12	59 ± 1	15.91 ± 0.44	750 ± 20	9.01
NR/A-Si-JF	1.77 ± 0.11	62 ± 2	17.04 ± 0.52	770 ± 20	10.88

**Table 4:** Temperatures at different stages of degradation of NR composite.

Formulation	Temperature (°C)			
	*T*_10%_	*T*_20%_	*T*_50%_	*T*_80%_
Unfilled NR	343	359	381	413
NR/JF_un_	341	359	382	420
NR/A-JF	335	355	381	417
NR/A-St-JF	338	357	382	419
NR/A-Si-JF	342	361	387	421
